# Protective effect of rosiglitazone on microscopic and oxidative stress parameters of ram sperm after freeze-thawing

**DOI:** 10.1038/s41598-022-18298-2

**Published:** 2022-08-17

**Authors:** Mahdieh Mehdipour, Hossein Daghigh-Kia, Abouzar Najafi, Zohreh Mehdipour, Hossein Mohammadi

**Affiliations:** 1 Department of Animal Science, College of Agriculture, University of Tabriz, Tabriz, Iran; 2 Department of Animal and Poultry Science, College of Aburaihan, University of Tehran, Tehran, Iran; 3 Prestage Department of Poultry Science, North Carolina State University, Raleigh, NC 27606 USA; 4 Department of Animal Science, Faculty of Agriculture and Natural Resources, Arak University, Arak, Iran

**Keywords:** Cytological techniques, Flow cytometry

## Abstract

The purpose of this study was to investigate the effects of rosiglitazone on ram semen after cryopreservation on the quality of thawed sperm. Sperm motility, membrane functionality, viability, total abnormality, acrosome membrane integrity, mitochondrial activity, reactive oxygen species production, ATP content and apoptotic features were assessed after thawing. Rosiglitazone at concentration of 60 µM resulted in the highest (*P* < 0.05) total motility, progressive motility and straight-line velocity. The percentages of average path velocity and curvilinear velocity were greater in the 60 µM group. Different concentrations of rosiglitazone did not have significant effects on amplitude of the lateral head displacement, linearity and straightness. The highest amounts of membrane functionality and mitochondrial activity after freeze-thawing were observed in groups containing 60 µM. By increasing the rosiglitazone level to 80 µM, no positive effect was observed in most of the evaluated parameters. The lowest ROS concentration was recorded in 60 µM rosiglitazone group (*P* < 0.05). The group containing 60 µM rosiglitazone also produced the lowest significant percentage of apoptosis-like changes and dead sperm. A greater (*P* < 0.05) percentage of acrosome integrity in frozen-thawed spermatozoa was observed in the 60 µM rosiglitazone group. There was no significant difference between 40 and 60 µM rosiglitazone in intact acrosome of ram thawed semen. The result showed that supplementation in ram semen extender with rosiglitazone had a positive role in the regulation of ram sperm motility and had strong protective effect on the sperm membrane and acrosome integrity.

## Introduction

Spermatozoa can be kept in liquid form for short periods of time or frozen for extended periods^1^. The only viable approach for long-term storage of biological material that ensures genetic integrity is cryopreservation. This strategy can be particularly beneficial for good broodstock management, endangered species conservation, and restocking natural populations of declining species^2^. Since this technique can cause significant sperm damage, it is critical to use cryoprotectants and antioxidants in cryopreservation solutions. The production of reactive oxygen species (ROS) and the alteration of oxidative metabolism during the freezing and thawing processes decrease the quality and viability of sperm^3^. The addition of antioxidants and drugs to freezing media could protect sperm from cryo-damage, counteracting the harmful effects of ROS. They have shown promising results in sperm cryopreservation^4^.

Rosiglitazone is categorized in a class of drugs known as ‘thiazoledinediones’ or ‘glitazones’, which sensitize the cells in the body to the action of insulin^5^. It is a PPAR‐γ (peroxisome proliferator-activated receptor-) agonist that has been shown to have antifibrotic and anti-inflammatory effects in some renal diseases^6^. Rosiglitazone's anti-inflammatory effects are closely linked to microglia polarization, which may be mediated by PPAR‐γ activation^7^. Rosiglitazone's main function is to reduce insulin resistance, but it also lowers LDL cholesterol levels in the blood, raises HDL cholesterol levels, causes minor changes in triglyceride levels, and lowers blood pressure. These rosiglitazone effects may even reduce the increased risk of cardiovascular disease in type 2 diabetes patients^8^.

Furthermore, numerous studies have shown that rosiglitazone improves skeletal muscle cell insulin sensitivity by increasing glucose uptake and muscle glycogen synthesis in mice fed a high fat diet^9,10^. Rosiglitazone regulates metabolic flexibility and glucose uptake in a variety of cell types and has received attention for its potential use in sperm production^1,11^. But its effects on ram sperm metabolism are unknown. In view of these facts, our hypothesis was to investigate if thawed ram spermatozoa may also benefit from rosiglitazone supplementation.

## Materials and methods

### Chemicals

All the chemicals were purchased from Merck (Darmstadt, Germany) company. Animal care protocols were performed in accordance with ARRIVE guidelines and the University of Tabriz guidelines for Animal Experiments. The Animal Research Committee of the University of Tabriz authorized the animal study.

### Semen collection

This research was carried out in research station and laboratory complex of Tabriz University. Four sexually mature Ghezel rams were sampled for sperm (3 to 4 years of age). Ejaculates were collected using an artificial vagina twice a week. The rams were in the same environmental conditions and nutrition.

### Semen processing and freezing

Immediately after semen collection, the samples were examined for volume, concentration and progressive motility, then the semen samples with a volume of 0.5–1.5 ml, motility above 80%, abnormal morphology less than 10% and sperm concentration above 3 × 10^9^ were used for the experiment. The volume of pooled semen was then extended at 37 °C with tris extender (1 part semen to 4 parts extender) comprising 7% (v/v) glycerol and 0 (control), 20, 40, 60 and 80 μM rosiglitazone. In this study, after diluting the semen, the gradual cooling step was performed in a refrigerator at 4° C for 2 h. Then, immediately after cooling the samples, they were aspirated into 0.25 ml French straws. In the next step, the straws were placed at a distance of 4 cm above the nitrogen surface. After 7 min, they were immersed in liquid nitrogen (-196° C) and were then transferred into a nitrogen tank^12^. For assessment, the cryopreserved straws were thawed in a water bath at 37 °C for 30 s.

### Sperm motility

A sperm analyzer (CASA) system IVOS 12 (Hamilton Thorne Inc., Beverly, USA) was used for analyzing motility parameters. At first, thawed sperm were diluted in PBS buffer before being placed on a prewarmed chamber slide (37 °C, Leja 4; 20 µm height; Leja Products, Luzernestraat B.V., Holland). Each sample had at least 200 cells examined. Total motility (TM, %), progressive motility (PM, %), average path velocity (VAP, µm/s), straight line velocity (VSL, µm/s), curvilinear velocity (VCL, µm/s), and linearity (LIN, %) were assessed^13^.

### Membrane functionality

In the present study, the test was performed according to the method of Najafi, et al.^14^ . Then, 10 μL of semen was added to 100 μL of hypoosmotic medium containing fructose and sodium citrate. It was then incubated at 37 °C for 30 min. At least three drops (10 μL) of the incubated sample were examined using a contrast phase microscope. Microscopic examination was performed using a hot plate at 37 °C with a magnification of 400. In each treatment group, at least 200 sperm were counted and the percentage of sperm with a tied tail was calculated.

### Abnormal sperm

To evaluate abnormal sperm, at least 3 drops of each sample were added to microtubes containing 1 mL of Hancock solution, and then one drop of this solution was placed on a slide and covered with a slide. Percentage of abnormal sperm was defined by counting at least 200 sperm under a microscope contrast phase at 400× magnification^15^.

### Assay of sperm viability and apoptosis

The percentages of viability, apoptotic and dead sperm were assessed by annexin kit. After thawing the semen samples, 500 μL of buffer was added and after centrifugation (1200 rpm for 10 min), the formed pellet was mixed with 500 μL of buffer. After mixing with 100 μL of calcium buffer, 10 μL of annexin was added and kept in a dark place for 20 min. Then, 10 μl of propidium iodide (PI) was added to the sample and incubated for 15 min. The amount of phosphatidylserine displacement of sperm membranes was then evaluated by flow cytometer. In the flow chart of the flow cytometer, if the samples are annexin negative and PI negative, they are considered as living sperm. If annexin is positive and PI is negative, the sample is alive but has primary apoptosis. If it is annexin positive and PI positive, it is considered as dead sperm with secondary apoptosis. Annexin negative and PI positive are considered as necrotic sperm^16^.

### Measurement of mitochondrial activity

In this study, mitochondrial activity was measured by rhodamine dye. At first, sperm samples were first mixed with 500 μL buffer. Then, 10 μL of rhodamine was added to the sample and maintained in dark at room temperature for 20 min. Then, 10 μL of PI was added to the sample and then the mitochondrial activity of the samples was measured by a flow cytometer. In the diagram provided by the flow cytometer, the rhodamine positive and PI negative samples are considered as active mitochondrial samples. If rhodamine is positive and PI is positive, it is considered as inactive mitochondria^17^.

### Determination of ATP in sperm

ATP was measured by the method of Kamali Sangani et al.^18^. At first, 5 µL of each sample was diluted in 750 µL of the buffer and 5 µL of the sample were pipetted to 190 µL of perchloric acid. Then centrifugation was carried out at 12,600×*g* for 2 min. The upper phase was transferred to another tube and 10.7 μL of 2 M KCl, 58.7 µL of 1 M KOH, 10.7 of saturated Tris and 1 µg/mL of red phenol were added. Finally, 100 µL luciferin–luciferase reconstituted reagent was added. Serial dilutions of the ATP standard were used to create standards with values ranging from 10^–7^ to 10^–12^ M. The amount of ATP was expressed as pmol ATP per 10^6^ sperm.

#### Determination of ROS

ROS measurement was done by the method of Kamali Sangani, et al.^18^. The samples were incubated for 20 min in 250 µL of the PBS at 37 °C. After centrifugation at 300×*g* for 7 min, the supernatant was removed. Then, 3 mL PBS was added, recentrifuged at 300×*g* for 7 min. Then, 10 µL of luminol was added to 400 µL of the sample and then the tubes were placed in an Orion II Microplate Luminometer. The results were expressed as 10^3^ counted photons per minute (cpm) per 10^6^ spermatozoa.

#### Assessment of acrosome membrane integrity

**Figure 1:**
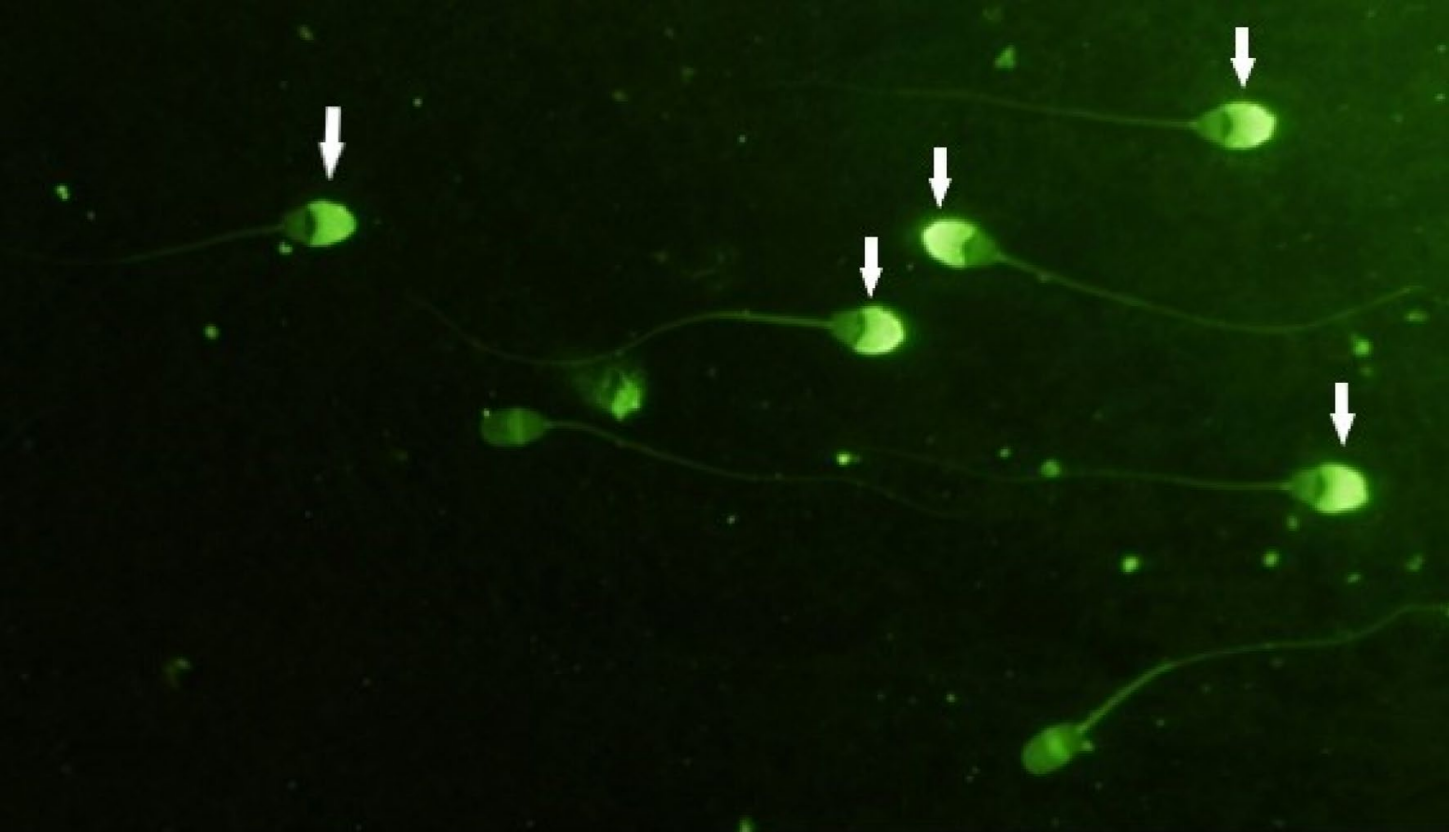
Example of spermatozoa as evaluated for acrosome integrity. Sperm that have completely absorbed the fluorescent green color in the head area are considered as sperm with healthy acrosome (Indicated by arrows) and sperm that did not absorb the fluorescent color or absorbed the color in limited areas are considered as acrosomes with damaged acrosome.

To perform this experiment, the straws were first thawed and the contents of each straw were centrifuged with 600 g for 10 min. Then the supernatant was removed and the pellet was dissolved in 100 μL of 96% ethanol and fixed for 4 min at 4 °C and then mixed by pipette. Then about 10 μL of the sample was placed on a slide and expanded using another slide and the ethanol was allowed to evaporate. Then 20 μl of PSA solution (50 μL / ml) was placed on the sample and a spread was prepared from it. The sample was then incubated for 15 min at 4 °C and immersed in distilled water, and dried. Then, glycerol was mounted on the sample and spread with a 24 × 24 mm slide. It was covered and then 200 sperm were counted on each slide using a fluorescent microscope with magnification of 400 samples. Sperm that completely absorb the fluorescent green color in the head area are considered as sperm with healthy acrosome and sperm that do not absorb the fluorescent color or absorbed the color in limited areas are considered as sperm with damaged acrosome (Fig. 1)^19^.

#### Statistical analysis

The results of the experiment were analyzed by SAS software using Proc MIXED. This experiment was performed in 8 replications. Differences were considered to be significant when their probability of occurring by chance was less than 5% (*P* < 0.05).

## Results

The effect of different concentration of rosiglitazone on motility parameters in freeze-thawed sperm is presented in Table 1. Rosiglitazone at concentration of 60 µM resulted in the highest (*P* < 0.05) total motility, progressive motility and VSL compared to the other groups. The percentages of VAP and VCL were greater in the 60 µM group as compared with the control, 20 and 80 µM rosiglitazone groups. The BCF of ram spermatozoa was greater in the 60 µM group as compared with the control group. There was no significant difference between 40 and 60 µM for VAP and VCL. Different concentrations of rosiglitazone did not have significant effects on the, ALH, LIN and STR.

**Table 1:** Results of the motility analysis (CASA).

Antioxidant	µM	TM (%)	PM (%)	VAP (µm/s)	VSL (µm/s)	VCL (µm/s)	LIN (%)	STR (%)	ALH (µm)	BCF (Hz)
Control		41.0 ± 2.4^c^	17.4 ± 0.6^c^	60.0 ± 1.6^c^	40.1 ± 0.2^c^	107.0 ± 1.7^b^	37.5 ± 0.5	67.2 ± 1.8	6.5 ± 0.18	19.6 ± 1.1^b^
Rosiglitazone	20	47.1 ± 1.6^c^	15.7 ± 1.1^c^	61.8 ± 1.7^bc^	41.3 ± 0.4^bc^	109.4 ± 1.4^b^	37.8 ± 0.7	67.1 ± 1.4	6.2 ± 0.12	20.4 ± 1.5^ab^
	40	58.8 ± 1.7^b^	25.3 ± 1.4^b^	65.0 ± 0.6^ab^	43.7 ± 0.9^b^	111.4 ± 2.4^ab^	39.5 ± 1.5	67.2 ± 1	6.2 ± 0.09	22.1 ± 1.5^ab^
	60	68.4 ± 1.5^a^	32.3 ± 1.2^a^	67.2 ± 0.5^a^	47.2 ± 1.3^a^	117.3 ± 1.8^a^	40.4 ± 1.4	70.3 ± 1.9	6.1 ± 0.16	25.6 ± 1.0^a^
	80	43.7 ± 2.6^c^	17.7 ± 1.5^c^	60.8 ± 0.7^bc^	41.0 ± 0.4^bc^	108.2 ± 06^b^	37.9 ± 0.3	67.6 ± 1.2	6.4 ± 0.06	19.7 ± 1.1^b^

*TM* total motility, *PM* progressive motility, *VSL* curvilinear velocity, *VAP* average path velocity, *VSL* straight-line velocity, *LIN* linearity, *STR* straightness, *ALH* amplitude of lateral head displacement, *BCF* beat/cross frequency. Different superscripts within the same column indicate significant differences among groups (P < 0.05).

**Figure 2:**
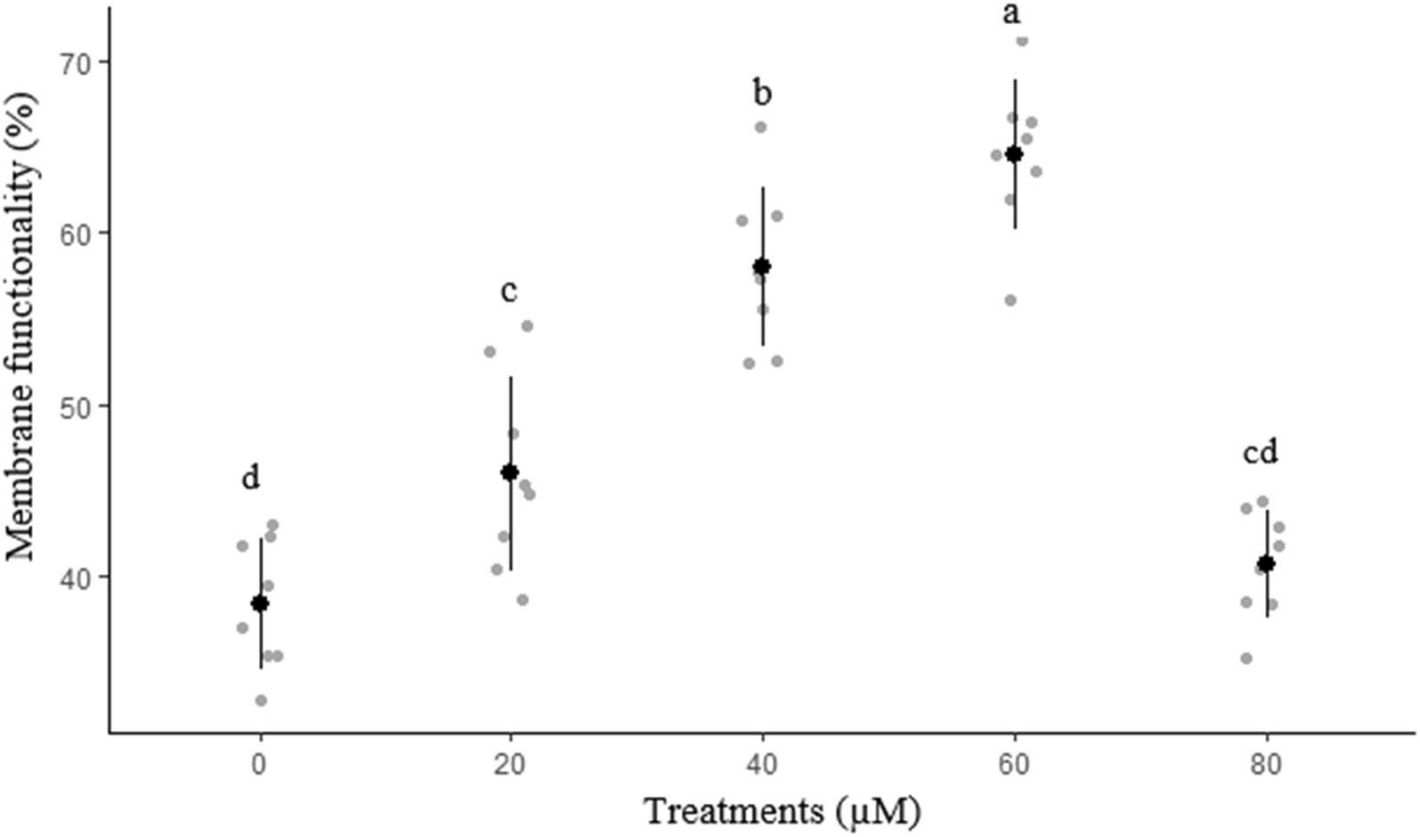
Effect of different levels of rosiglitazone on membrane functionality of ram sperm.

**Figure 3:**
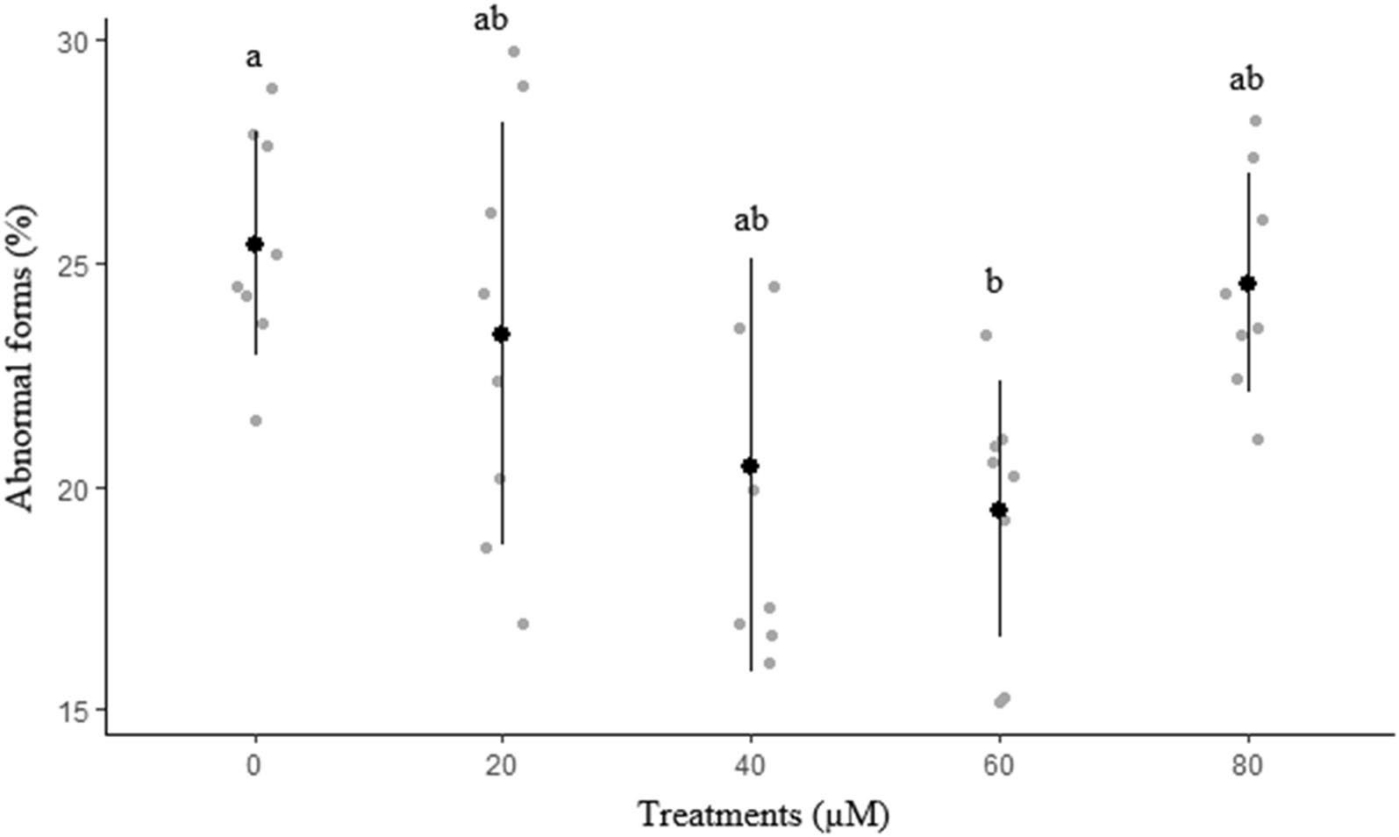
Effect of different levels of rosiglitazone on morphology status of ram sperm.

**Figure 4:**
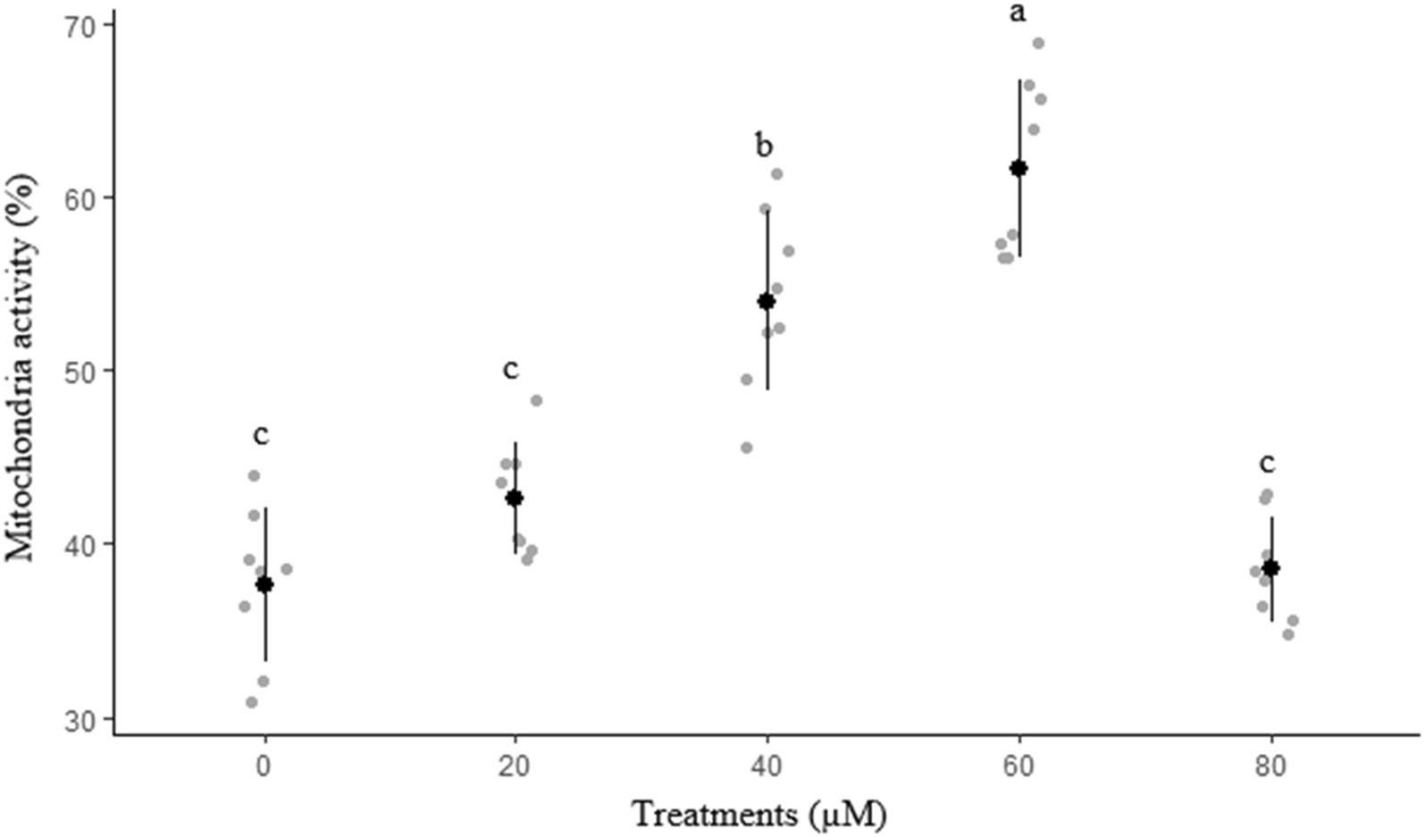
Effect of different levels of rosiglitazone on mitochondria activity of ram sperm.

**Figure 5:**
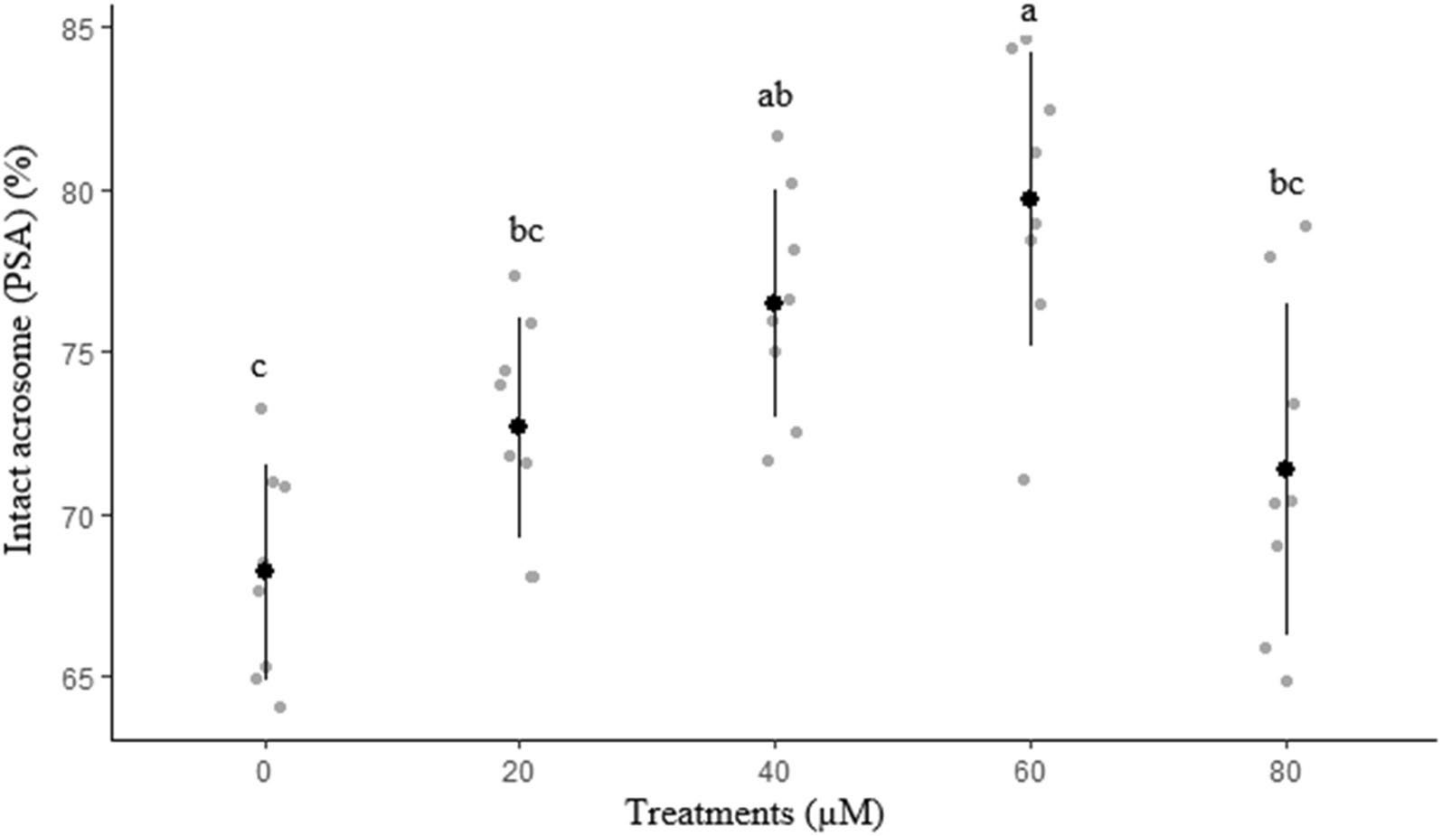
Effect of different levels of rosiglitazone on intact acrosome of ram thawed semen.

The effects of different concentrations of rosiglitazone on sperm membrane functionality, abnormal morphology, mitochondrial activity, and acrosome integrity are presented in Figs. 2, 3, 4 and 5, respectively. The highest (*P* < 0.05) amount of membrane functionality and mitochondrial activity after freeze-thawing were observed in groups containing 60 µM compared to the other groups. Based on the data, the minimum abnormality of sperm cells was observed in the group treated with 60 µM rosiglitazone. A greater (*P* ≤ 0.05) percentage of acrosome integrity in frozen-thawed spermatozoa was observed in the 60 µM rosiglitazone group when compared with the control.

**Figure 6:**
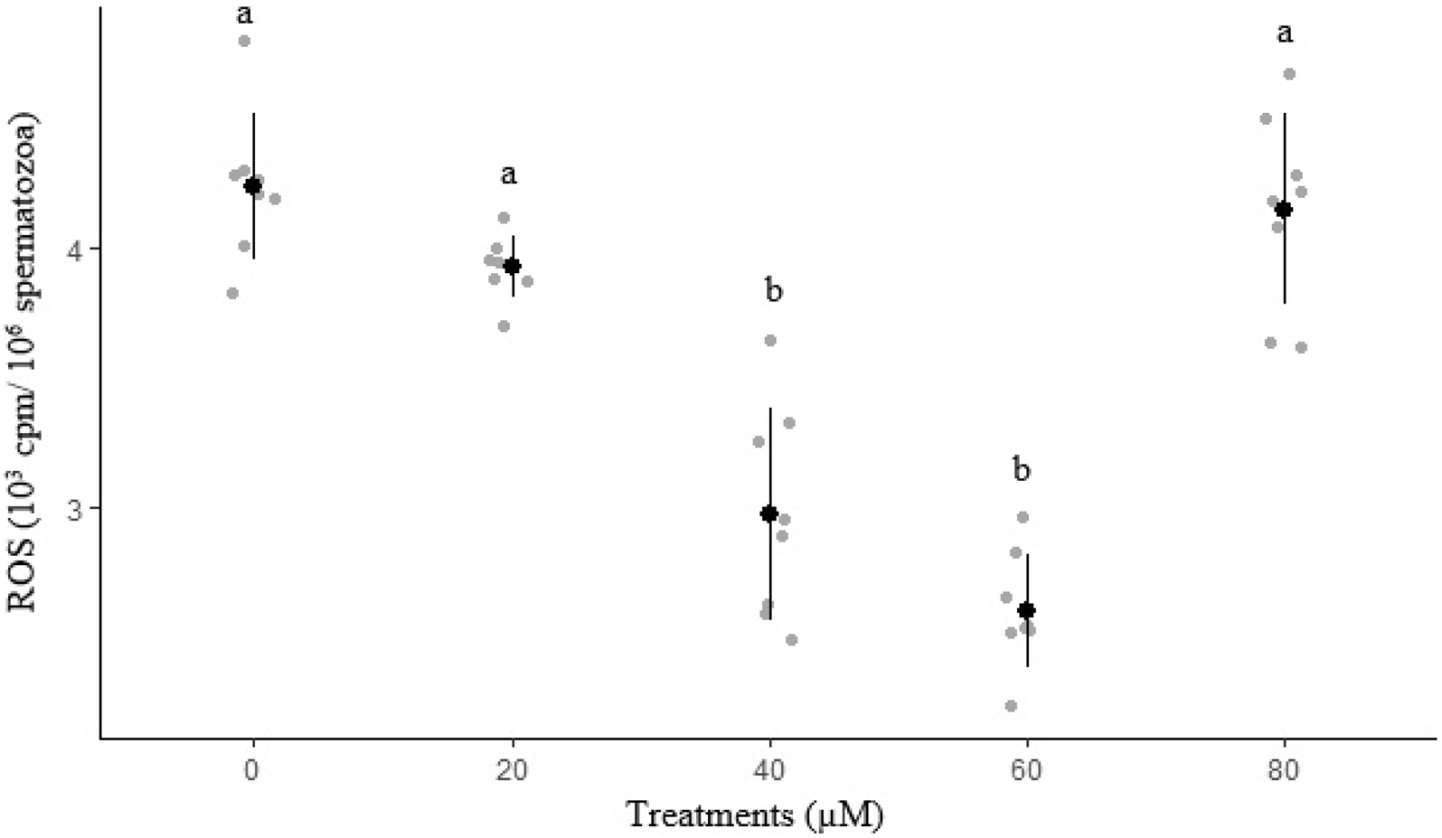
Effect of different levels of rosiglitazone on ROS of ram thawed semen.

**Figure 7:**
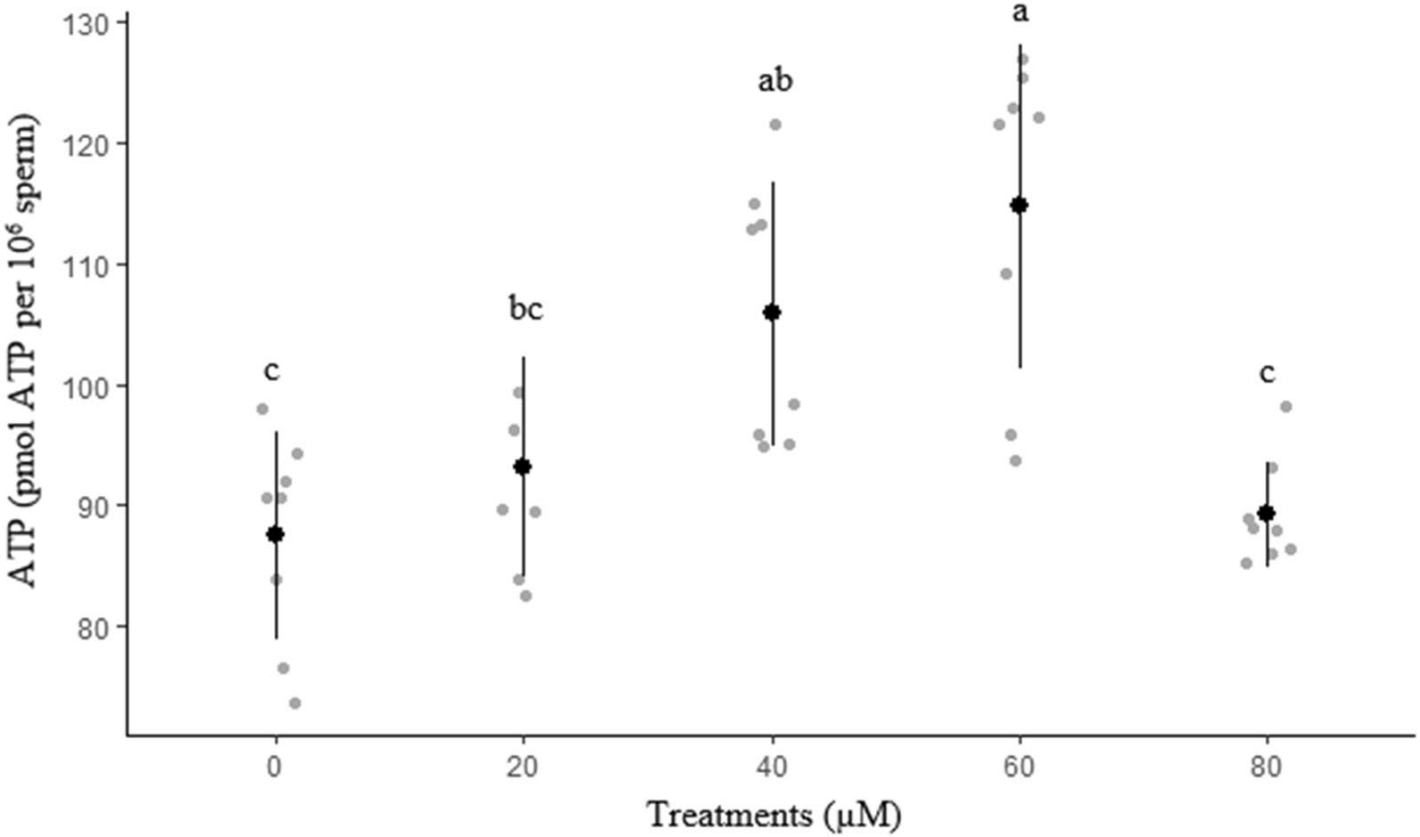
Effect of different levels of rosiglitazone on ATP of ram thawed semen.

Results of ROS and ATP in freeze-thawed sperm are shown in Figs. 6 and 7, respectively. The lowest (*P* < 0.05) ROS concentration was recorded in 60 µM rosiglitazone group. Group 60 µM rosiglitazone had a greater (*P*  ≤ 0.05) ATP level as compared with the control group.

**Figure 8:**
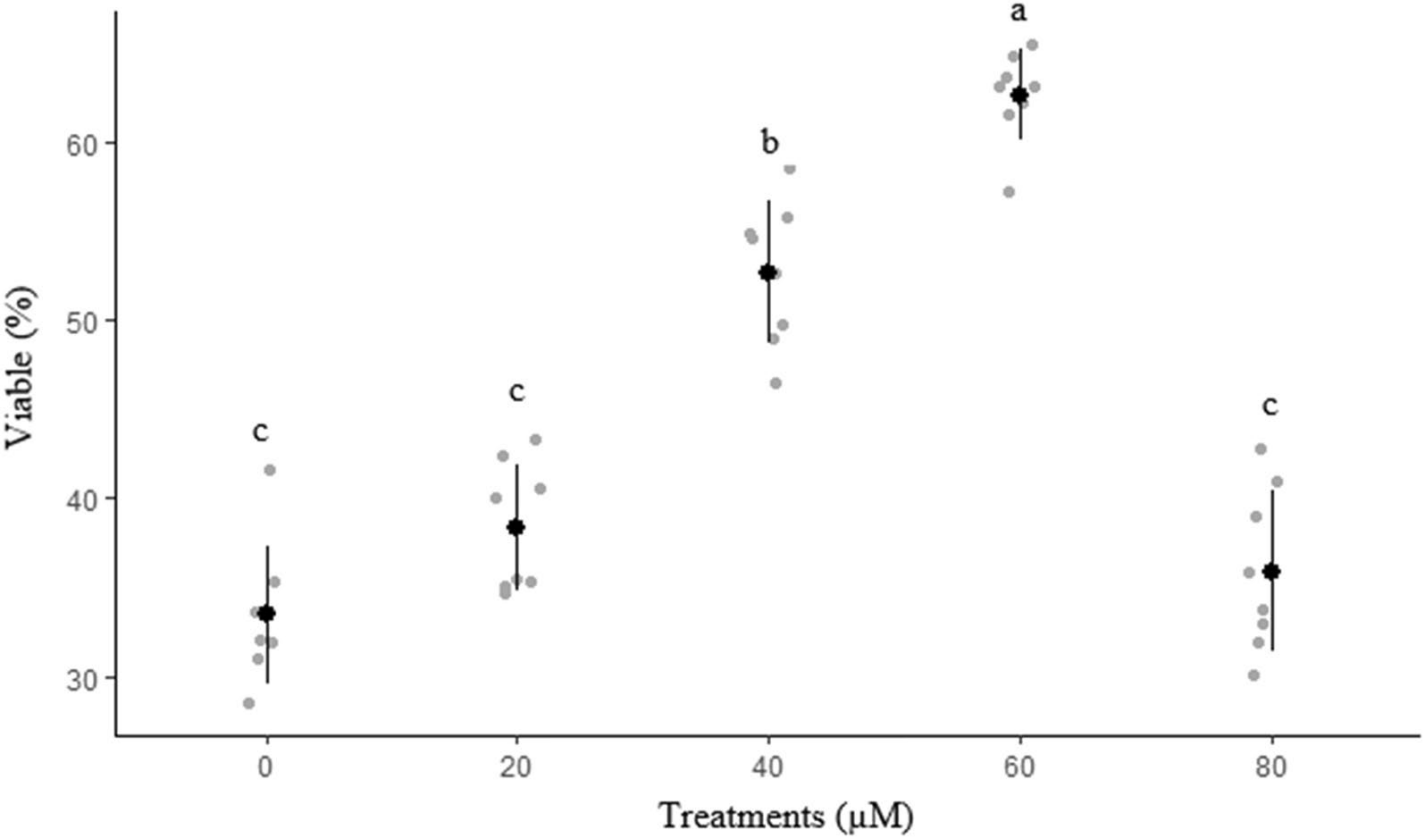
Effect of different levels of rosiglitazone on viability of ram thawed sperm.

**Figure 9:**
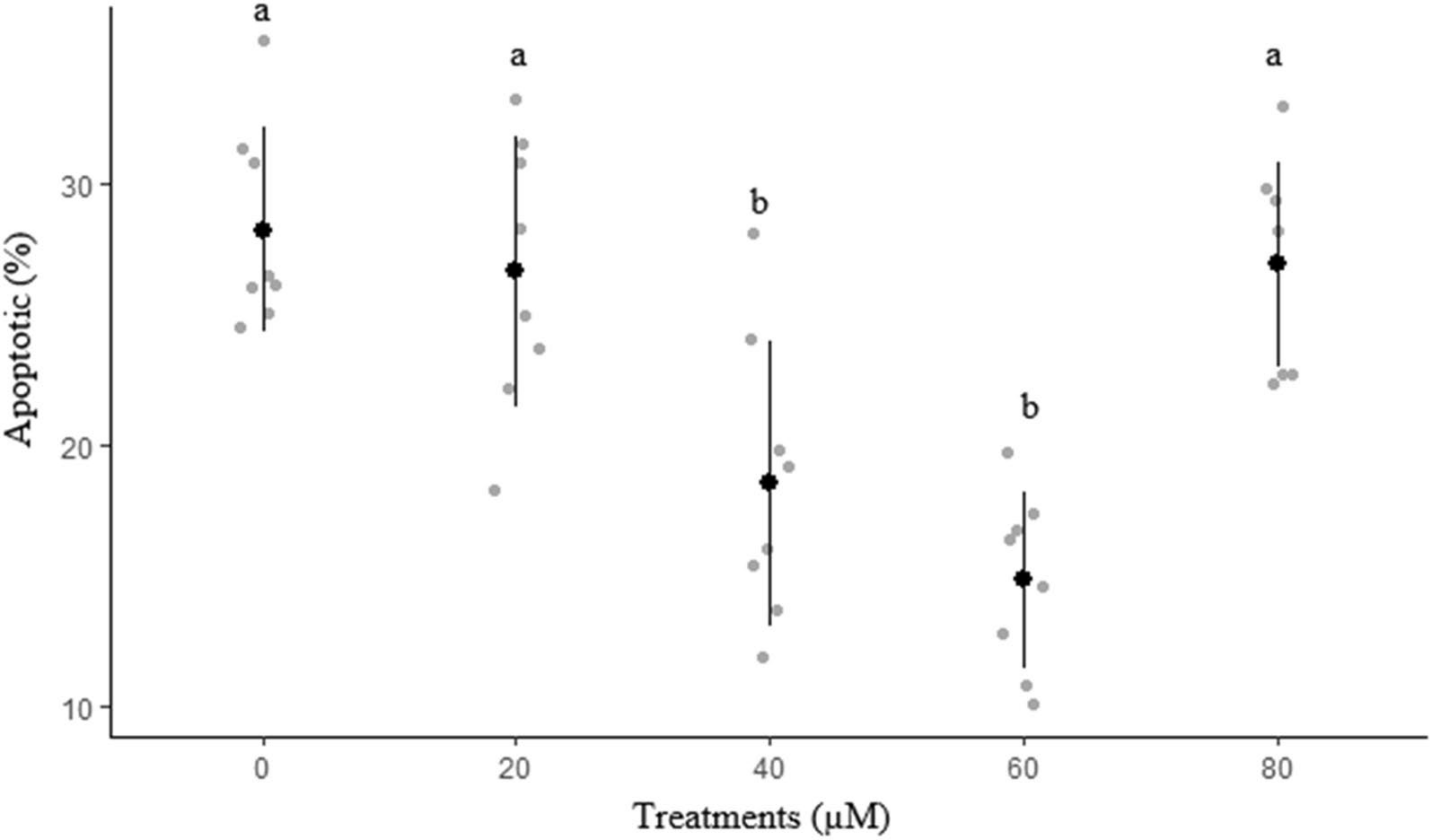
Effect of different levels of rosiglitazone on apoptotic of ram thawed sperm.

**Figure 10:**
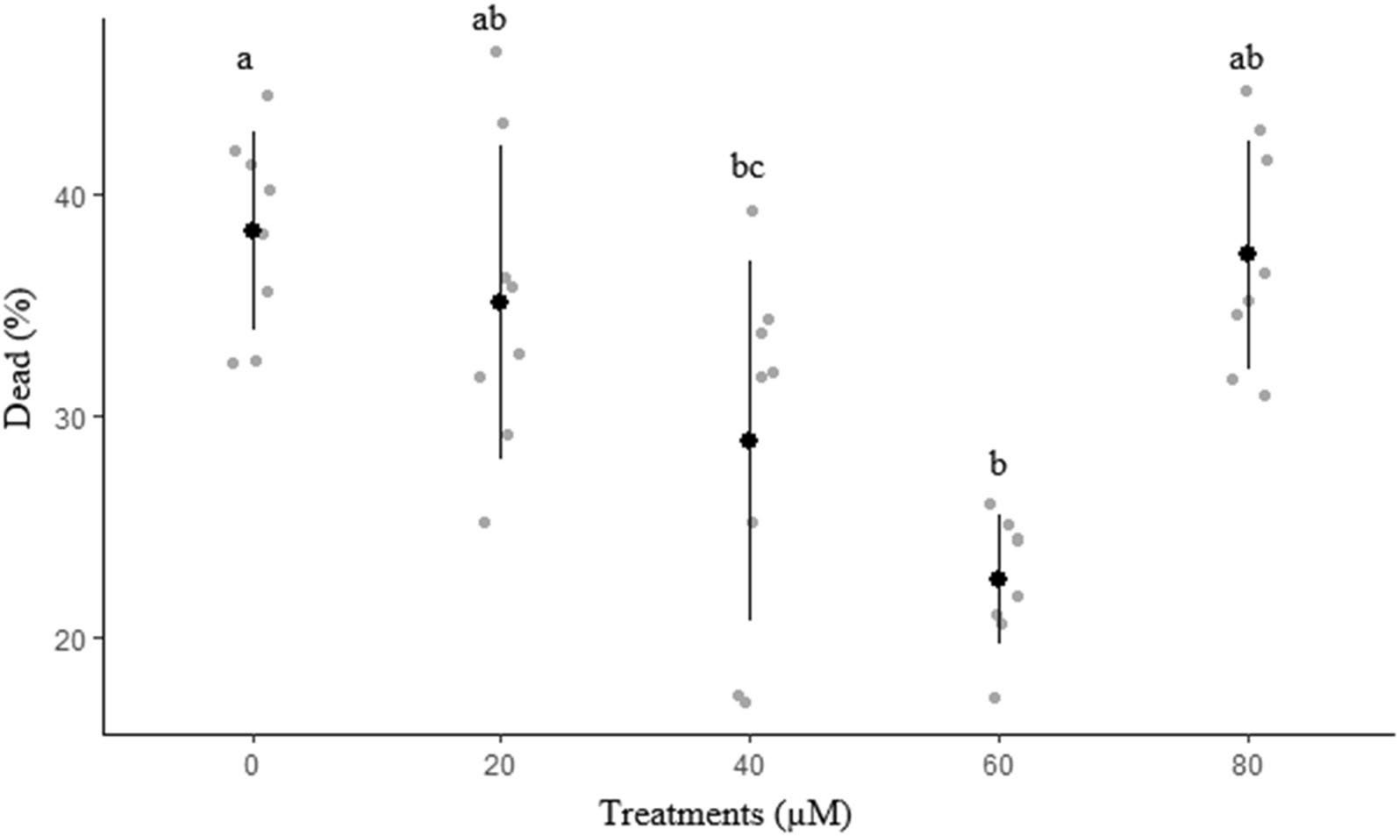
Effect of different levels of rosiglitazone on dead percentage of ram thawed semen.

Effects of different concentrations of rosiglitazone on viability and apoptosis-like changes of ram sperm after cryopreservation are shown in Figs. 8 and 9, respectively. The percentage of viable sperm was significantly higher in 60 µM rosiglitazone group compared to other groups (*P* < 0.05). It was also detected that 60 µM rosiglitazone group produced the lowest significant percentage of apoptosis-like changes and dead sperm (Fig. 10) compared to the other groups.

## Discussion

Sperm must pass through some procedures to become able to fertilize an egg and the process is facilitated by the release of certain enzymes from the sperm surface, which leads to specific binding of the surface-active component with the egg zona pellucida and final fusion of the two cells^20^. This interaction modifies the sperm surface. The sperm membrane is subjected to a series of extremely subtle and complex alterations throughout this process, including the elimination of suppression (decapacitation) factors and modifications to the lateral organization of the proteins and lipids on the sperm surface^21^. Semen cryopreservation is a beneficial approach for long-term storage of livestock sperm. This method alters several cellular physiochemical processes, which can lead to sperm damage.

Motility is usually the most dependable indicator of sperm fertility. During freezing and thawing, oxidative stress contributes to membrane lipid peroxidation and lowers sperm motility and viability^22^. Consequently, incubation of spermatozoa with rosiglitazone may protect membranes against oxidative damage. The current study showed that rosiglitazone has some favorable impacts on motility and velocity parameters, including TM and PM as well as VAP. These beneficial effects can be attributed to the regulating sperm energy metabolism, which protects the mitochondrial membrane potential^23^. This was revealed by the improvements in membrane integrity due to the addition of rosiglitazone to the extender, which improved post-thaw motion parameters. Other studies have found that rosiglitazone improves spermatozoa viability and motility. However, we can hypothesize that only the sperm with the best normal morphology remain viable under the effect of rosiglitazone after thawing and therefore are included in the pool recruited to be used for fertilization.

It is proved that rosiglitazone can increase the glycolytic activity of stallion spermatozoa kept at room temperature for long periods of time^1^. Furthermore, rosiglitazone has been shown to activate pro-survival pathways in spermatozoa in human and porcine studies^1,24,25^.

The HOST test measures the sperm plasma membrane resistance to damage caused by permeability loss during cryopreservation^26^. In this study, cryopreserved sperm samples with 60 µM rosiglitazone showed the highest sperm membrane functionality (HOST) and acrosome integrity which was similar to the results of Wang, et al.^23^. One of the functions of rosiglitazone in terms of acrosome integrity and membrane functionality is its ability to form a protective layer outside the sperm membrane when the sperm are subjected to cooling damage, thus reducing physical injuries after thawing of sperm^23^. This phenomenon may help sperm survive after cryopreservation by preventing damage. This improvement in sperm membrane integrity could lead to better sperm properties such as motility.

Mitochondria are essential for ATP synthesis, and any damage to them causes apoptosis. Cryodamage may be harmful to mitochondrial membrane potential (MMP) and ATP production. It is noteworthy that AMP-activated protein kinase (AMPK) has a critical role in energy balance, and sperm requires a specific level of AMPK activity to preserve an appropriate MMP^27^. According to a study, rosiglitazone also reduces NADPH-stimulated superoxide anion production in rat arteries, consequently decreases oxidative stress^28^. The generation of oxidative stress products, such as ROS, was significantly reduced as a result of our findings. The hydroxylation of the phenyl and pyridine rings in the chemical structure of rosiglitazone may enable the scavenging of hydroxyl radicals^29^. It has also been discovered that rosiglitazone's ability to reduce oxidative stress is mediated by inhibition of NAD(P)H oxidase. This effect causes by the inhibition of NO quenching by NAD(P)H oxidase–derived ROS^30^. The group supplemented by 60 μM rosiglitazone showed a decline in ROS content. This observation is also sustained by previous works which suggested that the ability of rosiglitazone to activate AMP-activated protein kinase (AMPK) is responsible for the majority of its antioxidative activity^31^. Therefore, it is proved that rosiglitazone reduces ROS primarily by inhibiting NAD(P)H oxidase through an AMPK-dependent mechanism^31^.

In the current study, supplementation of the semen extender with 60 μM rosiglitazone before cryopreservation improved ATP level. Mammalian sperm generates ATP primarily through two metabolic pathways, anaerobic glycolysis and oxidative phosphorylation (OXPHOS), locating in different parts of the cell. On the one hand, OXPHOS occurs in mitochondria, which are densely packed in the sperm mid-piece. It has been proved that in several mammalian species, OXPHOS has a supporting role as the chief ATP producer for sperm motility. Moreover, in these species, ATP content, motile sperm proportion, and sperm velocity are all positively related to MMP and oxygen consumption rate^32^. It is shown that boar sperm supplementation with rosiglitazone increased sperm glucose uptake capacity and lactate production. This suggests that rosiglitazone could enhance sperm glycolysis metabolism^33^. We hypothesize that the protective effect of rosiglitazone on the motility of sperm can be due to its effect on membrane functionality, acrosome integrity and mitochondrial potential. When compared to the control group, our findings showed that rosiglitazone increased mitochondrial membrane potential, increased ATP content, and decreased mitochondrial ROS generation in ram sperm. Our results are in concordance with Swegen, et al.^11^ who reported rosiglitazone could improve stallion sperm motility, ATP content, and mitochondrial function.

In the current study, sperm supplemented with 60 μM rosiglitazone had a lower rate of apoptosis while having a higher rate of viability and membrane functionality which is in line with Ortiz-Rodriguez, et al.^1^ . Pro-apoptotic factors releasing into the cytoplasmic compartment, and activating apoptotic pathways are caused by the opening of mitochondrial pores and decreases in membrane potential during sperm cryopreservation^34^. The thiols in spermatozoa are depleted during cryopreservation, resulting in an unstable redox status that quickly leads to redox deregulation. Caspase 3 is activated in this situation, resulting in sperm death. The present findings show that this type of sperm death can be postponed by rosiglitazone supplementation. In line with our study, it was reported in a research that addition of rosiglitazone to the samples increased higher percentage of live non-apoptotic spermatozoa after two hours of incubation^1^. The activation of metabolic flexibility is responsible for the positive effect of rosiglitazone supplementation on sperm viability in the current study. Therefore, spermatozoa may be more efficient at producing energy through glycolysis and fatty acid oxidation.

## Conclusion

The findings revealed that rosiglitazone supplementation in ram semen extender showed a positive role in the regulation of ram sperm motility and had a strong protective effect on sperm membrane, mitochondria activity, and acrosome integrity. Moreover, our findings showed that rosiglitazone increased ATP content, and decreased mitochondrial ROS generation in ram sperm.

## Supplementary Information

Supplementary Information.

## Data Availability

The authors declare that the data supporting the findings of this study are available within the paper.
